# Body Composition by DXA in Patients with Klinefelter and Kallmann Syndrome: The Kama Study

**DOI:** 10.1210/clinem/dgaf565

**Published:** 2025-10-10

**Authors:** Caterina Buoso, Andrea Delbarba, Matteo Riva, Giulia Artifoni, Elisa Gatta, Davide Farina, Eugenia Quiros-Roldan, Alberto Ferlin, Carlo Cappelli

**Affiliations:** Department of Clinical and Experimental Sciences, SSD Endocrinologia, University of Brescia, ASST Spedali Civili, Brescia 25121, Italy; Department of Clinical and Experimental Sciences, SSD Endocrinologia, University of Brescia, ASST Spedali Civili, Brescia 25121, Italy; Department of Medical and Surgical Specialties, Radiological Sciences and Public Health, University of Brescia, ASST Spedali Civili of Brescia, Brescia 25121, Italy; Department of Clinical and Experimental Sciences, SSD Endocrinologia, University of Brescia, ASST Spedali Civili, Brescia 25121, Italy; Department of Clinical and Experimental Sciences, SSD Endocrinologia, University of Brescia, ASST Spedali Civili, Brescia 25121, Italy; Department of Medical and Surgical Specialties, Radiological Sciences and Public Health, University of Brescia, ASST Spedali Civili of Brescia, Brescia 25121, Italy; Department of Clinical and Experimental Sciences, SD of Infectious and Tropical Diseases, University of Brescia and ASST Spedali Civili di Brescia, Brescia 25121, Italy; Unit of Andrology and Reproductive Medicine, Department of Medicine, University of Padova, Padua 35100, Italy; Department of Clinical and Experimental Sciences, SSD Endocrinologia, University of Brescia, ASST Spedali Civili, Brescia 25121, Italy

**Keywords:** Kallmann syndrome, Klinefelter syndrome, body composition, DXA

## Abstract

**Context:**

Klinefelter syndrome and Kallmann syndrome are 2 rare genetic disorders characterized by reduced testosterone (T) levels but differing in their gonadotrophin profiles. To date, no studies have directly compared body composition in these 2 syndromes.

**Objective:**

This work aimed to assess the prevalence of altered body composition parameters in patients with Klinefelter and Kallmann syndromes and to compare body composition between the 2 groups. Secondary objectives included evaluating associations between body composition, bone mineral density (BMD), and serum follicle-stimulating hormone (FSH) levels.

**Methods:**

This single-center, retrospective observational study included 50 patients, 29 with Klinefelter and 21 with Kallmann syndrome receiving T replacement therapy. Body composition was evaluated using whole-body dual-energy x-ray absorptiometry (DXA), which provided measurements of appendicular lean mass (ALM), total body fat (TBF), visceral adipose tissue (VAT), the ALM-to-height² ratio (appendicular lean mass index, ALMI), and the ALM-to-weight ratio.

**Results:**

Radiologic sarcopenic obesity was identified in 7 patients (14%; 6/29 Klinefelter, 1/21 Kallmann), while osteosarcopenic obesity was found in 2 patients (4%), both with Klinefelter syndrome. Patients with Kallmann syndrome had significantly higher ALMI values than those with Klinefelter syndrome (8.37 ± 1.15 vs 7.28 ± 1.20 kg/m²; *P* < .001). Univariable analysis revealed an inverse association between FSH levels and ALMI (B = −0.026; *P* = .002), which remained statistically significant after adjustment for confounders (B = −0.030; *P* = .0022).

**Conclusion:**

This study demonstrated a significant difference in lean mass between Klinefelter and Kallmann syndromes, supporting a potential role for FSH in modulating muscle mass independently of T levels.

The relationship between body composition and bone health represents an increasingly prominent area of research with potentially relevant clinical implications (1-3). Increasing evidence supports the influence of both muscle mass and body fat on bone mineral density (BMD) and bone quality. Bone, muscle, and adipose tissues communicate and interact with each other through endocrine and paracrine mechanisms. The term “*osteosarcopenic obesity*” has therefore been recently introduced to describe the coexistence of obesity in individuals with low BMD and reduced muscle mass. Osteosarcopenic obesity is associated with a higher risk of immobility, falls, fractures, and disability, and has been proposed as a potential clinical marker for predicting bone fragility (1, 3-5).

In individuals with hypogonadism, low testosterone (T) levels are well known to be associated with alterations in the bone, muscle, and fat tissue (6).

Klinefelter syndrome and Kallmann syndrome are 2 rare genetic conditions characterized by reduced T levels but differ in their gonadotrophin profiles, genetic backgrounds, and Leydig cell function. Klinefelter syndrome is the most common chromosomal disorder in males, typically characterized by the presence of one or more supernumerary X chromosomes. Clinical features are highly variable, although common characteristics are severely attenuated spermatogenesis and Leydig cell impairment, resulting in infertility and hypergonadotropic hypogonadism (7). Kallmann syndrome is a form of congenital genetic hypogonadotropic hypogonadism that includes anosmia or hyposmia as a distinguishing feature. It presents with absent or incomplete puberty and infertility, due to isolated gonadotrophin-releasing hormone deficiency in the context of otherwise normal anterior pituitary function (8). Altered body composition has been demonstrated both in Klinefelter and Kallmann syndromes, most likely due to hypogonadism as well as other conditions associated with each disorder (9-11). In addition, it has been reported that some Kallmann syndrome genes can have direct effects on bone (12, 13).

An expanding body of preclinical and clinical evidence seems to support the existence of extragonadal effects of follicle-stimulating hormone (FSH), particularly in the modulation of body composition, even if with critical gaps (14-19). In particular, FSH has been shown to affect bone metabolism through binding to specific receptors on osteoclasts. Elevated FSH levels have also been linked to increased adiposity and decreased muscle mass (18). Differences in BMD between individuals with hypergonadotropic and hypogonadotropic hypogonadism have already been described in the literature. However, the only available comparative study included patients with heterogeneous forms of hypogonadism, including nongenetic etiologies, and reported lower BMD levels in those with hypergonadotropic hypogonadism (17).

To date, no comparative studies investigating body composition parameters in patients with Klinefelter syndrome and Kallmann syndrome have been reported in the literature.

## Materials and Methods

### Patients

This single-center, retrospective observational study included male hypogonadal White patients on T replacement therapy (TRT) followed on an outpatient basis at the endocrinology unit of Spedali Civili di Brescia. In this study, the electronic medical records of patients older than 18 years diagnosed with Klinefelter syndrome or Kallmann syndrome, attending the outpatient clinics from January 1, 2020, to May 31, 2025, were analyzed. Inclusion criteria were (i) a verified Klinefelter syndrome karyotype (47, XXY) or (ii) hypogonadotropic hypogonadism associated with variable degrees of olfactory defects and genetically confirmed Kallmann syndrome (mutations in *ANOS1*, *FGFR1*, *PROKR2*, *GNRHR* genes).

Exclusion criteria included the absence of TRT and the use of osteoporosis medications, with the exception of vitamin D and calcium supplements. Patients with potential confounding factors, including anabolic or catabolic medications, relevant comorbidities such as diabetes mellitus or other chronic metabolic conditions, and extreme variations in exercise intensity, were excluded from the study. Kallmann patients with known pathogenic variants directly affecting bone metabolism were excluded (12, 13).

The primary outcomes were the prevalence of altered body composition parameters in individuals with Klinefelter and Kallmann syndromes, and the comparison of body composition between the 2 groups. Secondary outcomes included the associations between body composition, BMD, and serum FSH levels.

The study was approved by the local ethics committee (HYP-OS No. NP6062), and all participants provided written informed consent for the use of their clinical data for research purposes.

### Assessment of Body Composition

Body composition was assessed in all enrolled participants using total-body dual-energy x-ray absorptiometry (DXA), which provided measurements of appendicular lean mass (ALM, kg), total body fat (TBF, %), and visceral adipose tissue (VAT, cm²). Based on ALM, body size–adjusted indices were calculated, including the ALM-to-height² ratio (appendicular lean mass index, ALMI) and the ALM-to-weight ratio (ALM/W). “Reduced muscle mass” will be defined according to guideline-recommended cutoff values as the presence of ALM less than 20 kg (20), ALM/W less than 25.7% (21), and ALMI less than 7 kg/m² (20). “Increased adiposity” is assessed based on TBF% values, using cutoff points proposed in the literature for the male White population (>26% for ages 20-39; > 29% for ages 40-59; > 31% for ages 60-79 (21)), and on VAT, using the cutoff of greater than 130 cm² (5).

### Measurement of Bone Mineral Density and Vertebral Fractures Assessment

BMD was measured by DXA (Hologic Discover A) at the lumbar spine (LS), total hip (TH), and femoral neck (FN) in all enrolled participants. T-scores were used for participants aged 50 years or older, comparing BMD values with those obtained in a sex-matched White population at the peak of bone mass (22). In patients younger than 50, BMD was assessed using Z-scores, which compare individual values to those of an age- and sex-matched White reference population (22). For the purposes of this study, participants with a T-score below −1.0 or a Z-score below −2.0 were classified as having “low BMD.” Vertebral fractures (VFs) were identified on lateral spine x-rays through qualitative assessment of vertebral morphology and quantitative morphometric analysis.

### Biochemical Analyses

All blood samples were collected in the morning after an overnight fast. Total T (normal range, 2.49-8.36 mcg/L; limit of quantitation 0.12 ng/mL) levels were measured according to Endocrine Society Clinical Practice Guideline 2018: 2 to 8 hours after application of testosterone gel or prior to scheduled injections of T undecanoate (23). Circulating free T (cFT) was calculated based on total T, sex hormone–binding globulin (SHBG; normal range, 18.8-54.1 nmol/L; limit of detection 0.35 nmol/L), and albumin (normal range, 3.1-5.2 g/dL) levels according to the Vermeulen formula (24). Other hormonal parameters, including luteinizing hormone (LH; normal range, 1.7-8.6 IU/L; limit of quantitation 1 mIU/mL) and FSH (normal range, 1.5-12.4 IU/L; limit of quantitation 1 mIU/mL), were assessed using standardized assays. These hormonal assays were conducted using chemiluminescence-based techniques (Roche Diagnostics, Cobas E411 and E801 analyzers). The intra-assay and interassay coefficients of variation for T, FSH, LH, and SHBG were below 5%. Biochemical markers of calcium-phosphorus metabolism were also measured, including serum calcium (Ca; normal range, 8.6-10.2 mg/dL), phosphorus (P; normal range, 2.8-4.8 mg/dL), 25-hydroxyvitamin D (25OHD; sufficiency > 30 mcg/L), and parathyroid hormone (PTH; normal range, 15-65 pg/mL). Corrected Ca levels were calculated using the albumin-adjusted Ca formula: corrected Ca (mg/dL) = measured total Ca (mg/dL) + 0.8 × (4.0 – serum albumin [g/dL]). Additional routine blood tests evaluated included prostate-specific antigen (PSA; normal range <1.4 mcg/L), creatinine (normal range, 0.7-1.2 mg/dL), hemoglobin (Hb; normal range for male patients: 14-18 g/dL), and hematocrit (HCT; normal range, 42%-52%). All analyses were performed at the Central Laboratory of the University Hospital of Brescia.

### Statistical Analyses

Data are presented as mean ± SD for normally distributed variables, as median (interquartile range) for nonnormally distributed variables, and as categorical when appropriate. Normality was assessed using the Shapiro-Wilk test.

Continuous variables were compared using *t* test or the Mann-Whitney *U* test, as appropriate. Categorical variables were compared using the chi-square test or Fisher exact test, as applicable. Linear regression was employed to analyze the association between BMD, body composition parameters, FSH levels, and variables known to be relevant for bone health, after verifying the normality assumption of residuals, confirmed by the Shapiro-Wilk test and graphical analysis (histogram and Q-Q plot).

All statistical analyses were performed using SPSS 20.0 software (SPSS Inc) and R Studio 4.2.2. A statistical significance level of *P* less than or equal to .05 was set.

The results are reported in compliance with the STROBE reporting guidelines for cross-sectional studies; the checklist is reported in Supplementary file 1 (25).

## Results

A total of 57 patients were enrolled in the present study; 7 were excluded due to the use of antiresorptive therapy (4 individuals with Klinefelter syndrome and 3 patients with Kallmann syndrome). The final study population therefore consisted of 50 patients—29 (58%) with Klinefelter syndrome and 21 (42%) with Kallmann syndrome—with a mean age of 42 ± 16.3 years. Among the latter, 5 patients (17%) had undergone gonadotropin treatment for fertility induction in adulthood for up to 24 months. The clinical, biochemical, and anthropometric characteristics of the 2 groups are summarized in Table 1. The 2 groups of patients were comparable in terms of age at enrollment and diagnosis, smoking habits, body weight, body mass index, or general blood test parameters. Klinefelter and Kallmann patients significantly differed in FSH and LH serum levels (median 21.1 [16.1-30.6] vs 0.5 [0.5-0.5] IU/L; *P* < .001; and 40.8 [27.1-51.6] vs 0.5 [0.5-1.6] IU/L; *P* < .001, respectively) as expected. Eleven (22%) patients (9 of whom [82%] with Kallmann syndrome) were on therapy with T undecanoate, and 39 (79%) (27 of whom [69%] with Klinefelter syndrome) with T gel. Serum T was comparable (2.9 [2.7-3.8] vs 3.7 [2.9-4.4] mcg/L; *P* = .138) while TRT duration was higher in patients with Kallmann compared with those with Klinefelter (17 [8-32] years vs 6 [3-14.5] years; *P* = .023, respectively).

**Table 1.** Clinical characteristics of the 2 study groups

	Klinefelter (n = 29)	Kallmann (n = 21)	*P*
Age at study entry, y	41 (24-58)	41 (31-50)	.723
Age at diagnosis, y	28.3 ± 13.6	24 ± 9.86	.332
TRT duration, y	6 (3-14.5)	17 (8-32]	.**032**
No. of smokers	12 (41.4)	5 (23)	.210
Weight, kg	81 (65-95)	79 (72.5-92)	.976
Height, m	1.83 ± 0.09	1.76 ± 0.07	.**003**
WC, cm	96 (88-113)	90 (86-102)	.580
BMI	24.4 (20.8-27.7]	26.3 (24-28)	.154
LH, IU/L	21.1 (16.1-30.6)	0.5 (0.5-0.5)	**<**.**001**
FSH, IU/L	40.8 (27.1-51.6)	0.5 (0.5-1.6)	**<**.**001**
Type of TRT			
Undecanoate testosterone	2 (6.9)	9 (42.9)	.**003**
Gel testosterone	27 (93.1)	12 (57.1)	
Serum T, mcg/L	2.9 (2.7-3.8)	3.7 (2.9-4.4)	.138
Serum T in undecanoate T	3.4 (3.13-3.6)	4.4 (3-4.4)	.393
Serum T in gel T	3 (2.8-3.6)	3.1 (2.8-4.3)	.733
cFT, ng/L	51.5 (42.5-71.7)	75 (57.3-88.9)	.054
SHBG, nmol/L	52 (39.3-72.5)	35 (25.7-48.2)	.090
Ca, mg/dL	9.7 (9.5-10.1)	9.6 (9.5-9.7)	.204
Albumin-corrected Ca, mg/dL	9.8 (9.5-10)	9.6 (9.3-9.7)	.121
P, mg/dL	3.45 ± 0.67	3.02 ± 0.53	.076
PTH, pg/mL	39.8 (30.5-50.5)	46 (34.5-56.1)	.458
25OHD, ng/mL	35.3 ± 16.8	35.8 ± 13.5	.914
PSA, ng/mL	0.64 (0.40-0.85)	0.49 (0.29-0.76)	.319
Creatinine, mg/dL	0.93 (0.86-0.98)	1.01 (0.93-1.09)	.055
Hb, g/dL	15.1 ± 1.32	15.0 ± 1.03	.675
HCT, %	45.1 ± 3.51	44.6 ± 3.64	.637

Data are expressed as mean value ± SD, median (Q1-Q3) or absolute number (percentage). Statistically significant associations (*P* < .05) are highlighted.

Abbreviations: 25OHD, 25-hydroxyvitamin D; BMI, body mass index; Ca, calcium; cFT, calculated free testosterone; FSH, follicle-stimulating hormone; Hb, hemoglobin; HCT, hematocrit; LH, luteinizing hormone; P, phosphate; PSA, prostate-specific antigen; PTH, Parathyroid hormone; SHBG, sex hormone–binding globulin; T, testosterone; TRT, testosterone replacement therapy; WC, waist circumference.

### Bone Health

A total of 13 patients (26%) were found to have reduced BMD (9 with Klinefelter [31%] and 4 with Kallmann [19%]); vertebral deformities on morphometric assessment were identified in 4 patients (2 with Klinefelter [7%] and 2 with Kallmann [10%]). BMD values were compared both in absolute terms and as Z-scores, without revealing any statistically significant differences (Table 2). In the multivariable analysis—adjusted for potential confounding factors (serum T levels, age at enrollment, duration of TRT, smoking status, 25OHD levels, and albumin-corrected Ca levels)—a trend was observed for lumbar BMD (*P* = .069), with lower absolute values in patients with Klinefelter syndrome (1.003 ± 0.179 g/cm^2^) compared to those with Kallmann syndrome (1.057 ± 0.127 g/cm^2^) (Table 3).

**Table 2.** Altered body composition parameters in the overall cohort and in the 2 groups

	All participants (n = 50)	Klinefelter (n = 29)	Kallmann (n = 21)	*P*
Low BMD				
T-score ≤ −1.0 or Z-score ≤ −2.0	13 (26)	9 (31)	4 (19)	.340
Age <50 y	n = 33	n = 18	n = 15	
Z-score ≤ −2.0	4 (12.1)	4 (22.2)	0 (0)	.051
Age ≥ 50 y	n = 17	n = 11	n = 6	
T-score between −1.0 and −2.5	10 (58.8)	6 (54.5)	4 (66.7)	.627
T-score ≤ −2.5	0 (0)	0 (0)	0 (0)	
Reduced muscle mass				
ALM < 20 kg	9 (18)	7 (29.1)	2 (9.5)	.184
ALMI < 7 kg/m^2^	12 (24)	9 (31)	3 (14.3)	.171
ALM/W < 25.7%	8 (16)	6 (20.7)	2 (9.5)	.288
Increased adiposity				
Increased TBF (%)	24 (48)	16 (55.2)	8 (38.1)	.233
VAT >130 cm^2^	19 (38)	13 (44.8)	6 (28.6)	.309
Radiologic sarcopenic obesity				
Increased TBF and reduced ALM/W	7 (14)	6 (20.7)	1 (4.8)	.109
Radiologic osteosarcopenic obesity				
Increased TBF, reduced ALM/W and BMD	2 (4)	2 (6.9)	0 (0)	.219

Data are expressed as absolute number (percentage).

Abbreviations: ALM/W, Appendicular lean mass to body weight ratio; ALM, appendicular lean mass; ALMI, appendicular lean mass index; BMD, bone mineral density; TBF, total body fat; VAT, visceral adipose tissue.

**Table 3.** Comparison of dual-energy x-ray absorptiometry–derived parameters between the 2 study groups

	Klinefelter (n = 29)	Kallmann (n = 21)	*P*	*P* adjusted*^a^*
LS BMD, g/cm^2^	1.003 ± 0.179	1.057 ± 0.127	.245	.069
FN BMD, g/cm^2^)	0.821 ± 0.126	0.802 ± 0.120	.602	.886
TH BMD, g/cm^2^	0.984 ± 0.142	0.999 ± 0.134	.719	.351
LS Z-score, SD	−0.445 ± 1.717	−0.024 ± 1.302	.35	.156
FN Z-score, SD	−0.314 ± 0.980	−0.362 ± 0.923	.861	.730
TH Z-score, SD	−0.097 ± 0.996	0.0619 ± 0.979	.578	.300
ALM, kg	24.39 ± 4.74	25.64 ± 4.66	.362	.075
ALM/W, %	29.66 ± 4.06	31.38 ± 4.16	.148	.310
ALMI, kg/m^2^	7.28 ± 1.20	8.37 ± 1.15	.**002**	**<**.**001**
%BF, %	29.48 ± 7.75	25.59 ± 6.69	.070	.112
VAT, cm^2^	136.92 ± 77.94	118.43 ± 59.31	.368	.603

Data are expressed as mean value ± SD, median (Q1-Q3), or absolute number (percentage). Statistically significant associations (*P* < .05) are highlighted.

Abbreviations: 25OHD, 25-hydroxyvitamin D; ALM/W, appendicular lean mass to body weight ratio; ALM, appendicular lean mass; ALMI, appendicular lean mass index; BF, body fat; BMD, bone mineral density; FN, femoral neck; LS, lumbar spine; TH, total hip; TRT, testosterone replacement therapy; VAT, visceral adipose tissue.

^*a*^ *P* value after adjusting for age at study entry, TRT duration, smoking habit, serum testosterone, 25OHD, and albumin-corrected calcium levels.

The association between BMD and variables potentially relevant to bone health, including ALMI, TBF, VAT, serum FSH levels, and duration of TRT was explored in the entire cohort (Table 4). Both univariable and multivariable linear regression analyses were performed, with the latter adjusted for potential confounders such as age at enrollment, smoking status, and serum levels of T, 25OHD, and albumin-corrected Ca.

**Table 4.** Linear regression results for associations between bone mineral density (lumbar spine, femoral neck, total hip) and appendicular lean mass index, total body fat, visceral adipose tissue, follicle-stimulating hormone, and therapy duration

	*P*	B	95% CI	*P* adjusted*^a^*	B	95% CI
LS BMD						
ALMI	.095	0.03	−0.005 to − 0.065	.**028**	0.038	0.004 to 0.071
TBF	.836	0.001	−0.006 to 0.007	.351	−0.003	−0.010 to 0.004
VAT	.052	0.001	0.000 to 0.001	.489	0.000	−0.001 to 0.001
FSH	.063	−0.002	− 0.004 to 0.000	.073	−0.002	−0.004 to 0.000
TRT duration	.**007**	0.004	0.001 to 0.007	.612	0.001	−0.004 to 0.006
FN BMD						
ALMI	.309	0.014	−0.013 to 0.041	.251	0.016	−0.012 to 0.045
TBF	.9	0.000	−0.004 to 0.005	.764	−0.001	−0.007 to 0.005
VAT	.327	0.000	0.000 to 0.001	.159	0.001	0.000 to 0.001
FSH	.812	0.000	−0.001 to 0.002	.990	0.000	−0.002 to 0.002
TRT duration	.626	−0.001	−0.003 to 0.002	.965	0.000	−0.004 to 0.004
TH BMD						
ALMI	.**045**	0.030	0.001 to 0.060	.**044**	0.031	0.001 to 0.062
TBF	.292	0.003	−0.002 to 0.008	.608	0.002	−0.005 to 0.008
VAT	.**028**	0.001	0.000 to 0.001	.082	0.001	0.000 to 0.002
FSH	.612	0.000	−0.002 to 0.001	.553	−0.001	−0.003 to 0.001
TRT duration	.207	0.002	−0.001 to 0.004	.891	0.000	−0.004 to 0.005

Abbreviations: 25OHD, 25-hydroxyvitamin D; ALMI, appendicular lean mass index; BMD, bone mineral density; FN, femoral neck; FSH, follicle-stimulating hormone; LS, lumbar spine; TBF, total body fat; TH, total hip; TRT, testosterone replacement therapy; VAT, visceral adipose tissue.

Regression coefficients (B), 95% CI, and *P* values are presented for unadjusted models and models adjusted for potential confounders (*^a^*including testosterone levels, age at enrollment, smoking status, 25OHD, and albumin-corrected calcium levels). Statistically significant associations (*P* < .05) are highlighted.

**Figure 1.**
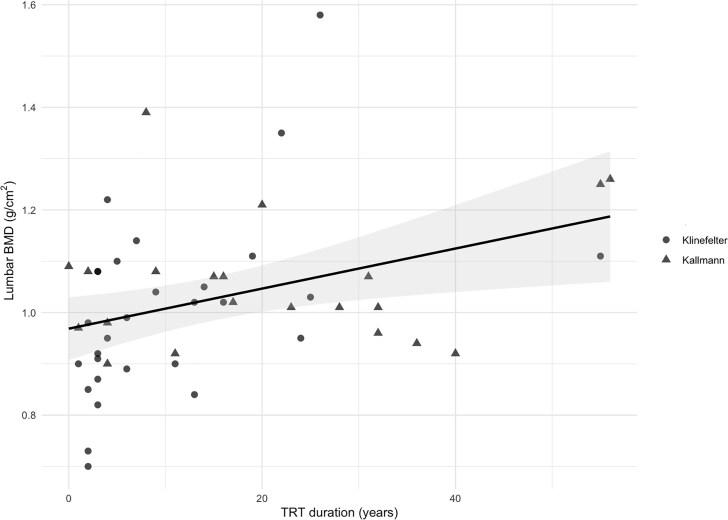
Scatterplot showing the univariable linear regression between lumbar bone mineral density (BMD) and testosterone replacement therapy (TRT) duration. The black line represents the fitted regression line, and the shaded area indicates the 95% CI of the estimate. Dots and triangles represent individual patients with Klinefelter and Kallmann syndromes, respectively.

**Figure 2.**
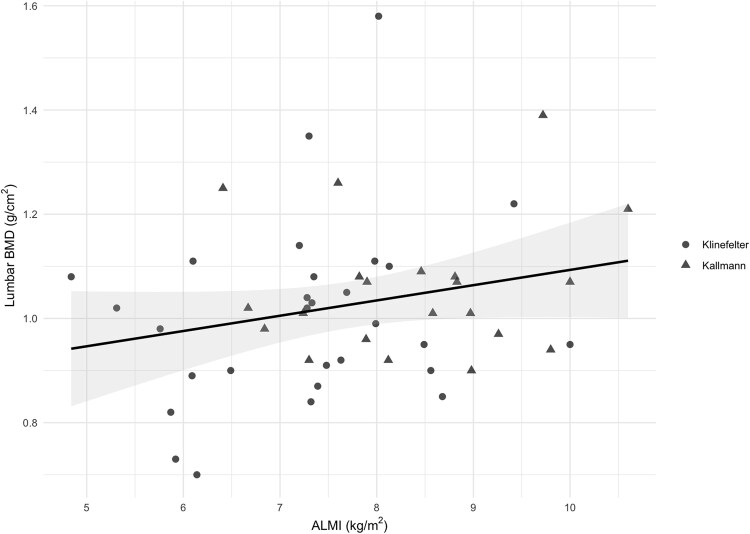
Scatterplot showing the univariable linear regression between lumbar bone mineral density (BMD) and appendicular lean mass Index (ALMI). The black line represents the fitted regression line, and the shaded area indicates the 95% CI of the estimate. Dots and triangles represent individual patients with Klinefelter and Kallmann syndromes, respectively.

Univariable analysis revealed a statistically significant association between TRT duration and lumbar BMD (B = 0.004; *P* = .007) (Fig. 1). However, after adjustment for potential confounding factors, this association was no longer statistically significant. Instead, an independent association between ALMI and lumbar BMD emerged (B = 0.038; *P* = .028) (Fig. 2). No statistically significant associations were found between the considered variables and femoral neck BMD. Regarding total hip BMD, ALMI remained a variable significantly associated with BMD in both univariable (B = 0.03; *P* = .045) and multivariable analyses (B = 0.031; *P* = .044). An association between VAT and total hip BMD was also observed in the univariable analysis (B = 0.001; *P* = .028), but this was not confirmed in the multivariable model (see Table 4).

### Body Composition

A total of 12 (24%) individuals showed a reduced muscle mass (based on ALMI values) whereas an increased TBF was observed in 24 (48%) and 19 (38%) showed an increase of visceral fat. The overall prevalence of radiologic sarcopenic obesity was found in 7 (14%) patients (6 with Klinefelter [21%] and 1 with Kallmann [5%]) whereas the prevalence of osteosarcopenic obesity was found in 2 (4%) patients, both of whom belonged to the Klinefelter group (see Table 2).

Patients with Kallmann syndrome showed higher ALMI values compared to patients with Klinefelter (8.37 ± 1.15 vs 7.28 ± 1.20 kg/m^2^; *P* < .001, respectively), at univariable analysis, whereas all the other parameters were superimposable between the 2 groups of patients (see Table 3). This difference remained statistically significant after adjustment for potential confounders (see Table 4).

**Figure 3.**
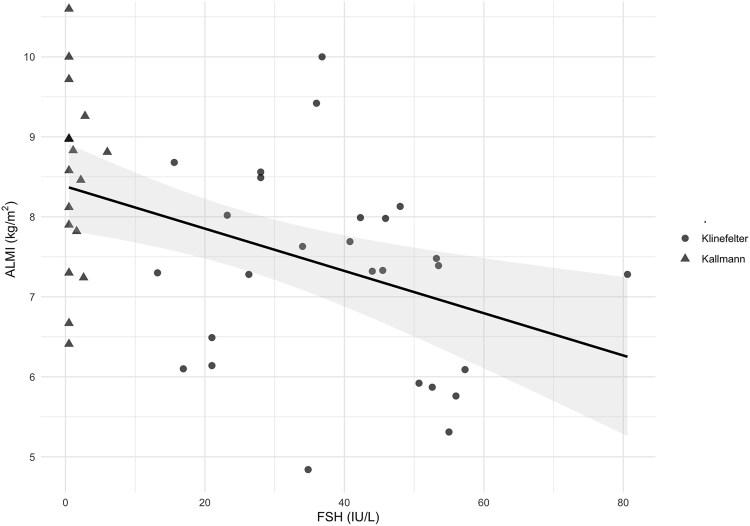
Scatterplot showing the univariable linear regression between appendicular lean mass Index (ALMI) and follicle-stimulating hormone (FSH) levels. The black line represents the fitted regression line, and the shaded area indicates the 95% CI of the estimate. Dots and triangles represent individual patients with Klinefelter and Kallmann syndromes, respectively.

Finally, the association between ALMI and potential determinants such as TBF, serum FSH levels, and duration of TRT was examined (Table 5). Univariable analysis showed an inverse association between FSH and ALMI (B = −0.026; *P* = .002), indicating a decrease in ALMI with increasing FSH levels (Fig. 3). This association remained statistically significant after adjustment for confounding factors (B = −0.030; *P* = .002).

**Table 5.** Linear regression results for associations between appendicular lean mass index and total body fat, follicle-stimulating hormone, and therapy duration

	*P*	B	95% CI	*P* adjusted*^a^*	B	95% CI
ALMI						
TBF	.497	0.017	−0.033 to 0.066	.816	0.008	−0.065 to 0.081
FSH	.**002**	−0.026	−0.043 to 0.010	.**002**	−0.030	−0.048 to 0.012
TRT duration	.545	0.008	−0.018 to 0.033	.745	−0.009	−0.063 to 0.045

Abbreviations: 25OHD, 25-hydroxyvitamin D; ALMI, appendicular lean mass index; FSH, follicle-stimulating hormone; TBF, total body fat; TRT, testosterone replacement therapy.

Regression coefficients (B), 95% CI, and *P* values are presented for unadjusted models and models adjusted for potential confounders (*^a^*including testosterone levels, age at enrollment, smoking status, 25OHD, and albumin-corrected calcium levels). Statistically significant associations (*P* < .05) are highlighted.

## Discussion

The present study showed no difference in BMD levels between patients with Klinefelter and Kallmann syndrome. However, for the first time, a significant difference in ALMI was identified, suggesting a role for FSH in regulating muscle mass independently from serum T levels and duration of TRT.

Klinefelter and Kallmann syndromes are 2 rare genetic conditions characterized by reduced T levels with different gonadotrophin profiles, genetic backgrounds, and Leydig cell function. Klinefelter syndrome is characterized by elevated gonadotrophin levels (7), whereas individuals with Kallmann syndrome display reduced FSH and LH concentrations (8). Despite for gonadotrophin levels, our patients were found to be largely homogeneous (see Table 1). A significant difference was observed in the duration of TRT, which was longer in the Kallmann group despite comparable age at diagnosis and age at study enrollment. This finding is also consistent with the clinical course of the 2 conditions: Individuals with Kallmann syndrome typically begin treatment at the time of pubertal induction, while in Klinefelter syndrome, hypogonadism may remain subclinical for years (7, 8). As a result, patients with Kallmann syndrome have a longer cumulative exposure to T, which may partly account for differences in bone and muscle outcomes. In addition, the trend toward a difference in cFT levels (*P* = .054) was observed between the 2 groups pf patients. This could be related to differences in treatment formulation or adherence rather than reflecting true biological differences between the groups.

Among our patients, the prevalence of reduced bone mineralization was found to be similar to that reported in previous studies on hypogonadal patients (9, 26), without a difference between individuals with Kallmann and Klinefelter syndrome. In addition, a similar prevalence of VFs has been shown.

In addition, no differences in BMD values were observed between patients with Klinefelter and Kallmann syndrome (see Table 3). Although the difference in lumbar BMD between the 2 groups showed only a trend toward statistical significance, the absolute difference was small (∼0.05 g/cm²) and is unlikely to be clinically relevant at the individual level, suggesting at most a modest effect that warrants confirmation in larger cohorts. This finding differs from that reported by Giovanelli et al (16), who showed a significant reduction in lumbar BMD in hypergonadotropic patients. We must underline that these data are not fully comparable with ours because Giovanelli and colleagues (16) also included patients with acquired hypogonadism who were not receiving TRT and had a later diagnosis. In addition, other hormones, such as estradiol, and/or different lifestyle may influence our findings. Notably, multivariable analysis, taking into account all potential factors influencing bone health, showed that only ALMI values were associated with BMD values at both lumbar spine and total hip (see Table 4). However, we underline that the regression coefficients were 0.038, raising questions about its clinical relevance. Regardless, this finding seems to confirm the positive effect of muscle on bone, a relationship that is well known (1-3, 5).

Body composition evidenced an overall prevalence of radiologic sarcopenic obesity in 7 (14%) patients (6/29 Klinefelter and 1/21 Kallmann), whereas the prevalence of osteosarcopenic obesity appeared in 2 (4%) Klinefelter individuals (Table 2). The prevalence of reduced lean mass—assessed using the ALMI—was 3- to 4-fold higher than reported in the general male population (24% vs 3.8%) (27).

By comparing ALMI values between the 2 study groups, we showed for the first time a significant reduction of muscle mass in patients with Klinefelter syndrome compared to those with Kallmann syndrome— even after adjustment for potential confounding factors (see Tables 3 and 4). This difference may be attributable to multiple factors. For instance, we found a significant inverse correlation between ALMI and FSH levels both in univariable and multivariable analyses (see Fig. 3, Table 5). Again, as previously shown for ALMI and BMD, the regression coefficient was small (−0.030).

The BLADE study, by Bergamini et al (16), investigating the relationship between circulating FSH levels and body composition, in prostate cancer patients undergoing androgen deprivation therapy, reported that higher FSH levels were associated with increased fat mass and a reduced ALMI-to-fat mass ratio after 12 months of androgen deprivation therapy. Similar findings were confirmed by Park et al in late perimenopausal women (28). We confirm and extend, for the first time, the relevance of FSH in influencing muscle mass in genetically hypogonadal male patients.

However, other hypotheses regarding the complex interplay between the musculoskeletal system and gonadal function should also be considered. In Klinefelter syndrome, for example, the typical testicular damage impairs Leydig cell function, which—as described in the literature—may negatively affect bone health and body composition through reduced insulin-like 3 (INSL3) secretion and altered vitamin D metabolism (9). INSL3, a well-established biomarker of Leydig cell function, also exerts anabolic effects on the osteomuscular unit (29, 30). In addition, vitamin D deficiency in these patients can also be secondary to lifestyle factors and/or sequestration in adipose tissue (26, 31). Additionally, genetic factors specific to each syndrome may further contribute to the observed differences: In Klinefelter syndrome, greater impairment in genetic expression, androgen deficiency, and reduced androgen receptor sensitivity have been associated with a more severe clinical presentation (32, 33). In addition, the role of estradiol should also be considered. Estradiol plays a key role in male skeletal maturation during puberty and in the maintenance of BMD in adulthood (34). Men with congenital hypogonadotropic hypogonadism are known to have markedly reduced circulating estradiol concentrations, which correlate with decreased LH serum levels (35, 36). Furthermore, recent evidence has demonstrated a positive association between ALMI and serum estradiol levels both in men living with HIV and postmenopausal women (37, 38).

Sarcopenia is a well-recognized condition associated with adverse clinical outcomes such as physical disability, reduced quality of life, and increased mortality (20, 21, 39). A potential clinical implication is that early intervention targeting reduced muscle mass may be beneficial in these patients. Nonpharmacological strategies, including physical activity and nutritional support, could enhance muscle function and in turn improve BMD (40).

To our knowledge, this is the first study to provide an estimate of the prevalence of radiological sarcopenic obesity and osteosarcopenic obesity in these patient populations. Currently, data on the prevalence of these 2 conditions in the general population are limited, partly due to the lack of universally accepted diagnostic cutoffs. Therefore, large-scale cohort studies are needed to allow for accurate comparisons.

Moreover, based on our findings, it would be of interest to further investigate patients with Klinefelter syndrome—both hypogonadal and eugonadal—in order to clarify the effect of hormonal status on body composition. Indeed, all our patients were in a state of pharmacologically induced eugonadism, which precluded assessment of the direct effects of hypogonadism on bone health and body composition but allowed evaluation of the effects of TRT itself.

One limitation of this study is the small sample size, which reflects the rarity of the syndromes under investigation. Further research involving larger cohorts is needed to validate our findings. Moreover, due to the small number of patients with vertebral deformities (n = 4), we were unable to evaluate the relationship between body composition and fracture risk. Such analysis would be of particular value for identifying additional determinants of fracture risk in secondary osteoporosis, for which BMD alone has limited predictive power. This could support a more personalized approach to bone health assessment, potentially in conjunction with conventional fracture risk tools.

It would also be of interest to explore bone microarchitecture in these patients using techniques such as the trabecular bone score, which offers improved fracture risk estimation in secondary osteoporosis. Furthermore, our study did not include functional tests for sarcopenia (eg, handgrip strength or chair stand tests), which would have complemented the radiological findings by providing functional evidence of muscle impairment.

A further limitation of our study is that circulating levels of INSL3 and estrogens were not assessed. An increasing body of evidence underscores the pivotal role of estrogens not only in female bone health but also in the male skeleton—potentially exerting an even greater influence than T (41). Meanwhile, INSL3, beyond its role as a Leydig cell biomarker, is known for its anabolic effects on the osteomuscular unit (29). Therefore, future studies should therefore consider evaluating these biochemical parameters to better understand their effect on body composition and bone health in patients with Klinefelter and Kallmann syndromes.

Although we did not collect detailed data on lifestyle components—particularly nutrition and physical activity—all patients reported adherence to a Mediterranean diet based on their medical history. Moreover, none of the patients had comorbidities that would have limited their ability to engage in physical activity, making it unlikely that low levels of physical activity were unevenly distributed between the 2 groups. Nonetheless, as physical activity levels were not objectively assessed, we cannot exclude the possibility that differences between the groups may have influenced the results.

### Conclusion

This study represents the first direct comparison of body composition between patients with Klinefelter and Kallmann syndrome—2 distinct and emblematic forms of hypogonadism, characterized by hypergonadotropic and hypogonadotropic profiles, respectively. While no statistically significant difference in BMD was observed between the 2 groups, this is the first study to demonstrate a significant difference in lean mass between these 2 genetic conditions. Furthermore, this study supports a possible role for FSH in regulating muscle mass, and a connection between muscle mass and bone health was highlighted in this specific patient population.

The lack of a significant difference in BMD may be partially attributed to the small sample size, but also to additional factors such as the mitigating effects of TRT on FSH-driven mechanisms, as well as the distinct genetic backgrounds of the 2 syndromes, which may influence body composition independently of T levels. In patients with these conditions, early lifestyle interventions—alongside pharmacological treatment—should be encouraged. A multidisciplinary approach incorporating muscle-strengthening exercise and nutritional support may offer added benefits for improving bone health in this population.

## Data Availability

Original data generated and analyzed during this study are included in this published article.

## Abbreviations

25OHD: 25-hydroxyvitamin D
ALM/W: appendicular lean mass to body weight ratio
ALM: appendicular lean mass
ALMI: appendicular lean mass index
BF: body fat
BMD: bone mineral density
Ca: calcium
cFT: circulating free testosterone
DXA: dual-energy x-ray absorptiometry
FN: femoral neck
FSH: follicle-stimulating hormone
HCT: hematocrit
INSL3: insulin-like 3
LH: luteinizing hormone
LS: lumbar spine
P: phosphorus
PSA: prostate-specific antigen
SHBG: sex hormone–binding globulin
PTH: parathyroid hormone
T: testosterone
TBF: total body fat
TH: total hip
TRT: testosterone replacement therapy
VAT: visceral adipose tissue
VF: vertebral fracture
